# Circadian variation in MGMT promoter methylation and expression predicts sensitivity to Temozolomide in glioblastoma

**DOI:** 10.21203/rs.3.rs-7411649/v1

**Published:** 2025-08-29

**Authors:** Maria F. Gonzalez-Aponte, Yitong Huang, William A. Leidig, Tatiana Simon, Omar H. Butt, Marc D. Ruben, Albert H. Kim, Joshua B. Rubin, Erik D. Herzog, Olivia J. Walch

**Affiliations:** 1 Department of Biology, Division of Biology and Biomedical Sciences, Washington University in St. Louis, St. Louis, MO, 63130, USA; 2 Department of Mathematical Sciences, Smith College, MA, 01063, USA; 3 Department of Neurosurgery, Washington University School of Medicine, St Louis, Missouri, USA; 4 Division of Oncology, Department of Medicine, Washington University School of Medicine, St Louis, Missouri, USA; 5 Divisions of Pulmonary Medicine and Biomedical Informatics, Cincinnati Children’s Hospital, USA; 6 The Brain Tumor Center, Siteman Cancer Center, Washington University School of Medicine, St Louis, Missouri, USA; 7 Department of Pediatrics, St. Louis Children’s Hospital, Washington University School of Medicine, St. Louis, MO, 63110, USA; 8 Department of Neuroscience, Washington University School of Medicine, St. Louis, MO, 63110, USA; 9 Arcascope Inc., Arlington, VA, 22203, USA; 10 Department of Neurology, University of Michigan, Ann Arbor, MI, 48104, USA

**Keywords:** GBM, TMZ, MGMT expression and promoter methylation, Chronotherapy, Chronodiagnosis

## Abstract

**Purpose:**

Recent studies show that glioblastoma (GBM) is more sensitive to Temozolomide (TMZ) in the morning. In cells, inhibiting O6-Methylguanine-DNA-Methyltransferase (MGMT) abolished time-dependent TMZ efficacy, suggesting that circadian regulation of this DNA repair enzyme underlies daily TMZ sensitivity. Here, we tested the hypotheses that MGMT-promoter methylation and protein abundance vary with time-of-day in GBM, resulting in daily rhythms in TMZ efficacy.

**Methods:**

We assessed daily rhythms in *Mgmt*-promoter methylation in GBM *in vitro* and retrospectively analyzed MGMT methylation status in human GBM biopsies collected at different times of day. Next, we measured MGMT and BMAL1 protein abundances in GBM cells collected at 4-hour intervals. To understand the therapeutic implications of circadian variations in MGMT, we incorporated its daily rhythms into an *in vitro* mathematical model capturing interactions between MGMT, TMZ, and GBM DNA.

**Results:**

We found daily rhythms in *Mgmt*-promoter methylation and protein levels in GBM *in vitro,* and in patient biopsies peaking at midday. Further, MGMT protein levels peaked at CT4, corresponding to the time of maximal TMZ efficacy *in vitro*. When we incorporated cell-intrinsic circadian rhythms in MGMT protein into a mathematical model for GBM chemotherapy, we found that dosing when daily MGMT levels peaked and began to decline produced maximum DNA damage.

**Conclusion:**

Our findings suggest that the likelihood of diagnosis of MGMT-promoter methylation varies with time of biopsy in GBM. Furthermore, we predict that efforts to deliver TMZ after the daily peak of MGMT activity, with exact time being dose-dependent, will significantly enhance its therapeutic efficacy.

## Introduction

Glioblastoma (GBM) is the most common primary malignant brain cancer with more than 300,000 adults diagnosed globally each year and a median survival of 15–18 months post-treatment [1, 2]. The current standard of care for GBM involves maximal safe surgical resection of the tumor, followed by concurrent radiation and chemotherapy with Temozolomide (Temodar^®^, TMZ), and tumor-treating fields [3, 4]. TMZ was introduced into the standard of care for GBM in 2005, and was found to induce the death of GBM cells by methylating O6-guanine, N7-guanine, and N3-adenine bases in DNA [5], leading to an extension of 2.5 months in patient overall survival [6, 7]. Unfortunately, response to TMZ is not universal, as the most frequent TMZ-induced cytotoxic lesion, O6-methylguanine (O6-MeG), can be repaired by the DNA repair enzyme O6-Methylguanine-DNA Methyltransferase (MGMT) in tumors that express this protein [5]. Thus, the presence or absence of MGMT is considered a prognostic factor for GBM patients, as tumor cells with epigenetic silencing of the *Mgmt* promoter (i.e., MGMT-methylated) are more susceptible to DNA damage than those that express *Mgmt* (i.e., MGMT-unmethylated) [8–10]. Based on this, prevailing clinical practice suggests that GBM patients with truly unmethylated *Mgmt* promoter show limited benefit from TMZ treatment [11]. However, recent work has revealed that *Mgmt* promoter methylation is not static. Rather, GBM cells have intrinsic daily fluctuations in *Mgmt* promoter methylation and by extension, sensitivity to TMZ [11]. This led us to hypothesize that determining MGMT methylation status may be biased by biopsy time-of-day.

Rhythms in *Mgmt* methylation and gene expression [12] suggest that MGMT protein may vary over the course of the day and thus, treatment efficacy may be improved with dosing timed in accordance with MGMT activity. Evidence for this can be found in a single-institute retrospective clinical study, in which delivering TMZ in the morning (AM) increased overall survival by 6 months, compared to evening (PM) treatment, particularly in GBM patients with *Mgmt*-methylated tumors [13]. Specifically, the effect sizes of AM vs. PM dosing in patients with *Mgmt*-methylated tumors are comparable (>2 months increase in overall survival) to the effect size of AM dosing vs. not taking TMZ at all, suggesting that dosing at the wrong time of day could have close to no benefit for these patients [13]. These findings were recently recapitulated in cellular and orthotopic models of GBM, where dosing with TMZ at the daily peak of circadian clock gene *Bmal1* expression significantly increased tumor cell death [13–15]. Importantly, time-dependent sensitivity to TMZ was abolished when treating GBM tumors with an inhibitor of MGMT, suggesting that daily expression of MGMT modulates sensitivity to chemotherapy with TMZ [14]. A recent retrospective analysis also found time-of-day effects of TMZ on progression-free survival in MGMT-unmethylated GBM patients [16]. That study differed in their definition of the morning group (i.e., midnight to 11am) and failed to find time-of-day effects in MGMT-methylated patients and on overall survival [16]. These results motivate us to better understand the underlying mechanisms of TMZ efficacy, and the role played by daily rhythms in MGMT expression and activity.

Here, we test the hypotheses that rhythms in *Mgmt* promoter methylation and protein expression regulate time-dependent TMZ vulnerability and can influence methylation status diagnosis. We find the likelihood of detecting *Mgmt* promoter methylation in human biopsies peaks around midday, suggesting methylation status diagnoses may be biased by the time of sample collection. Further, we find that MGMT protein abundance peaks in the early subjective morning in GBM cells *in vitro,* at Circadian time 4, near the minimum of *Mgmt* gene expression and at that time when TMZ efficacy was found to be higher in previous studies [13–15]. To understand why maximum TMZ efficacy occurred when delivered in phase with maximum MGMT protein abundance, we incorporate the observed daily activity of MGMT into an *in vitro* mathematical model and find that TMZ-induced DNA damage is maximized by dosing as MGMT levels are near their peak and beginning to decrease, with best times dependent on dose. Altogether, our findings suggest that optimal TMZ-induced cell death occurs when dosing in the subjective early morning.

## Materials and Methods

### Experimental methods

#### - Glioblastoma cell culture

LN229 cells, a female human GBM cell line obtained from the American Type Culture Collection, were cultured as a monolayer in coated T-75 flasks (Nunclon Delta coated, Fisher) using DMEM/F12+GlutaMax (Thermo Fisher) supplemented with 5% fetal bovine serum (FBS, Fisher) and 1% Pen/Strep (Thermo Fisher). Cells were maintained in a 5% CO2 incubator at 37°C. Passage number in all experiments ranged from six to twelve.

Glioma 261 cells (GL261, obtained from the Division of Cancer Treatment and Diagnosis Tumor Repository of the National Cancer Institute), a male murine model of GBM, were cultured in monolayer in coated T-75 flasks (Nunclon Delta coated, Fisher) using RPMI-1640 (Sigma-Aldrich) supplemented with 10% FBS (Fisher), 1% L-Glutamine (Thermo Fisher), and 1% Pen/Strep (Thermo Fisher). Cells were maintained in a 5% CO2 incubator at 37°C. Passage number in all experiments ranged from six to twelve.

Nf1^−/−^DNp53 male astrocytes (generous gift of Dr. Joshua Rubin, Washington University in St. Louis), a syngeneic murine model of GBM, were cultured in monolayer in coated T-75 flasks (Nunc Treated EasYFlasks, Fisher) using 10mL DMEM/F12 media (Gibco), supplemented with 10% FBS (Fisher) and 1% Pen/Strep (Thermo Fisher). Cells were grown in a 37°C incubator with a 5% CO2 environment. Passage number in all experiments ranged from five to ten.

Primary human B165 (MGMT methylated, male, generous gift of Dr. Albert Kim, Washington University in St. Louis) were cultured as spheres in 100mm uncoated petri dishes (Fisher) using 3mL DMEM/F12 Glutamax media (Gibco), supplemented with 1% Pen/Strep (Thermo Fisher), 2% B27 (Miltenyi Biotec), 25mg/10mL Heparin (Sigma-Aldrich), 20mg/100ml EGF (Pepro Tech), and 2mg/100ml bFGF (Pepro Tech). Cells were grown in a 37°C incubator with a 5% CO2 environment. Passage number in all experiments ranged from six to ten.

#### - Bmal1 knockdown

To knockdown *Bmal1* in LN229 cells, a predesigned shRNA plasmid packaged into a lentivirus (vector pLKO.1) was obtained from Sigma Aldrich (target sequence: GCAGAATGTCATAGGCAAGTT). Cells were grown in T-25cm^2^ flasks for 24h and incubated for 10 minutes at 37°C in 3mL complete DMEM media with 10% FBS (Thermo Fisher), 5% Pen/Strep (Thermo Fisher), and 15mg polybrene (Millipore #TR-1003-G). Following incubation, 5–10mL of virus stock solution was added to each culture. Media was changed after 24 h and cells were kept at 37°C, 5% CO2. Infected cells were selected using puromycin (1.5 mg/mL, Thermo Fisher) for 10 days. Knockdown efficiency was quantified by qPCR.

#### - Quantitative real-time PCR (qRT-PCR) and Quantitative Methylation Specific PCR (qMSP)

Genomic DNA (gDNA) was extracted and purified from 500,000 cultured LN229, LN229-Bmal KD, B165, and Nf1^−/−^DNp53 GBM cells collected at CT4 or CT16 using the DNeasy Blood and Tissue Kit (Qiagen), according to the manufacturer’s instructions. The purified gDNA underwent bisulfite conversion using the EZ DNA Methylation-Gold Kit (Zymo Research), following the protocol provided by the manufacturer. The concentration and purity of gDNA were measured at each step using a NanoDrop spectrophotometer (Fisher Scientific). Gene expression changes were further probed using iTaqTM Universal SYBR Green Supermix (Bio-Rad). Beta-actin was used as an internal control. Universal primers recognizing both methylated and unmethylated Beta-actin sequences were used. The following primer sequences were used: Methylated MGMT forward 5′-TTTCGACGTTCGTAGGTTTTCGC-3′, Methylated MGMT reverse 5′-GCACTCTTCCGAAAACGAAACG-3′, Unmethylated MGMT forward 5′-TTTGTGTTTTGATGTTTGTAGGTTTTTGT-3′, Unmethylated MGMT reverse 5′-AACTCCACACTCTTCCAAAAACAAAACA-3′, Beta-actin forward 5′-CTTCGCGGGCGACGAT-3′, and Beta-actin reverse 5′-CCACATAGGAATCCTTCTGACC-3′. qPCR amplification was carried out at 40 cycles with 10 ng of bisulfite treated gDNA in triplicates. Protocol is as follows: Cycle 1: 95 °C for 3 min; Cycle 2: 95 °C for 30 s; Cycle 3: 60 °C for 30 s; repeat step 2–3 for 39 more times; Cycle 4: 72 °C for 1 min. Negative controls included no template DNA samples. All procedures were done in triplicate in three biological replicates.

#### - Immunostaining and Microscopy analysis

Cells were grown in 35mm dishes containing a glass coverslip and fixed every 4h for 24h, starting 24h after initial plating, using 4% PFA for 10 minutes. Circadian time (CT) was defined relative to expression of the clock gene *Per2*, where CT0 corresponded to peak *Per2* expression [14, 17]. Cells were permeabilized for 30 minutes with 3% Triton-X (Millipore Sigma) in 1x PBS, and blocked for 1 hour with solution containing 10% BSA (Sigma) and 0.3% Triton-X. Mouse anti-MGMT (1:500, Invitrogen, Waltham, MA) and rabbit anti-BMAL1 (1:500, Abcam, Cambridge, MA) were diluted in 2% blocking solution and incubated overnight at 4°C. Samples were then rinsed three times with 1x PBS and incubated in secondary antibody solution (1:500 Alexa 488 goat anti-mouse IgG, and 1:500 Alexa 647 donkey anti-rabbit IgG, Abcam, Cambridge, MA) in 2% blocking solution for 1 hour at RT. Samples were rinsed 3 times in PBS, stained with ProLong Gold mounting medium with DAPI (Life Technologies, Carlsbad, CA), and stored in darkness at 4°C until imaging. To ensure specificity of the primary antibodies binding to the antigen and the secondary antibody binding to the primary, we included control samples with no primary or secondary antibodies, respectively. Each experiment was done in triplicate, in two biological replicates. Six imaging frames were collected and averaged per dish. Microscopy analysis was performed using ImageJ software. Quantification of protein expression was performed as corrected total cell fluorescence (CTCF) = Integrated Density – (Area of selected cell * Mean fluorescence of background readings), as described by McCloy et. al (McCloy et al., 2014).

#### - In vitro cell growth assays and pharmacology with daily TMZ

GBM cells were plated and grown for 48 hours to allow for attachment and growth. Cells were then treated with one of three TMZ concentrations (10, 100, 1000mM) or vehicle (DMSO, 0.2%), at either Circadian time 4 (CT4) or CT16. Circadian time was defined relative to expression of the clock gene *Per2*, where CT0 corresponded to peak *Per2* expression [14, 17]. Cells were fixed after 72h with 4% paraformaldehyde (PFA) and stained with 4′,6-diamidino-2-phenylindole (DAPI, 2mg/mL). We chose to measure survival 3 days after TMZ administration to allow for approximate 2–3 cycles of cell division and TMZ-induced DNA lesions, as done previously [14]. DAPI fluorescence was quantified with the Infinite 200 PRO plate reader (V_3.37_07/12_Infinite, Tecan Lifesciences). We calculated the percent of cell death as 100 minus percent cell survival (percent cell survival = number of living cells treated with TMZ divided by the number of living cells treated with vehicle). All procedures were done in triplicate.

#### - Statistical analysis

The Jonckheere-Terpstra-Kendall (JTK) cycle algorithm, a non-parametric test used to distinguish between rhythmic and non-rhythmic data, was used to assess circadian rhythmicity in MGMT protein expression with a level of p < 0.05 used to designate significant circadian rhythmicity [18]. Statistical significance of mean differences was determined by either Student’s t tests or two-way analysis of variance (two-way ANOVA) with multiple comparisons and Šídák’s post hoc test. All statistical analyses were performed in Prism (version 10.3.0).

### Retrospective analysis of Mgmt promoter status in GBM biopsies

To determine if *Mgmt* promoter methylation status varied by time of day in human GBM patients, we performed a retrospective chart review on patients diagnosed with GBM at Barnes-Jewish Hospital/Siteman Cancer Center. Inclusion criteria included all patients with newly diagnosed WHO Grade IV GBM (IDH-wildtype), diagnosed from January 2020 to October 2024 to ensure reliable determination of IDH status and *Mgmt* promoter methylation status. A total of 302 patients met these inclusion criteria. Their clinical and demographic variables, such as *Mgmt* promoter methylation status, age, sex, and time of surgery were collected. Time of surgery was determined by the recorded end time of anesthesia to approximate when the tissue samples were embedded for analysis. *Mgmt* methylation status was determined by bisulfite PCR profiling of the *Mgmt* promoter sequence. JTK cycle was used to assess circadian rhythmicity in *Mgmt* promoter methylation status, with a level of p < 0.05 used to designate significant circadian rhythmicity [18].

### Modeling methods

To simulate the effects of TMZ on tumor DNA *in vitro*, the following system of differential equations was used:

$$\frac{\text{dD}}{\text{dt}}=k_{1}Z\left(1-D\right)-k_{2}\text{MD}\;\frac{\text{dM}}{\text{dt}}=-\alpha \;\text{sin}\left(t\pi /12\right)$$

In this cell-based model [19], *Z* represents TMZ toxin (MTIC) concentration, *M* represents MGMT protein concentration, and *D* represents the fraction of damaged DNA in tumor cells *in vitro*. We compared results from this model to a simplified model in which TMZ was modeled as an impulse followed by exponential decay (see supplemental materials for details, Figure S3-S6). Additionally, exploratory simulations were carried out using a modified equation for DNA damage in which a Michaelis-Menten term is added (Figures S7-S9), which are also described in the supplemental materials. The results from the cell-based model without the Michaelis-Menten term are presented in the main text and figures.

In this system of equations, *Z* creates damaged DNA *D* at rate *k*_1_, in proportion to the amount of undamaged DNA, 1 − *D*. The parameter *k*_2_ removes damaged DNA in proportion to the amount of available MGMT and damaged DNA, *MD*. We modified the original model to include circadian variations in *M* (i.e., MGMT protein) as a sinusoid with a period of 24 hours, with initial conditions chosen such that it reaches its maximum value 4 hours after the start of the simulation, circadian time 4 (CT4), in keeping with experimental data. The MGMT derivative scalar *α* and initial condition *M*_0_ were chosen to make it so the maximum value of *M* occurs at *t* = 4 (CT4) and a 10-fold change in *M* occurs over the course of the day. Finally, we assume that the initial conditions for *Z* and *D* are *Z*_0_ = 0 and *D*_0_ = 0, respectively. This reduces our unknown parameters at baseline to *k*_1_ and *k*_2_, corresponding to the relative strengths of TMZ at inducing DNA damage and MGMT at repairing it.

#### Cell death

To model TMZ-induced apoptosis, we assume that the likelihood of cell death is proportional to the total DNA damage over time; ∫ *D*(*t*)*dt* = ***D***. We also assume that DNA damage exceeding threshold *D*_*T*_ kills all cells. This fatal threshold *D*_*T*_ can be solved for uniquely using data that shows that 1000 μM TMZ demonstrates approximately 60% as much variation in dosing efficacy over the course of the 24 hour day as 100 μM TMZ [14].

This *in vitro* model makes the following assumptions: 1) The only factor affecting the impact of TMZ is the presence or absence of MGMT, 2) the apoptosis decision is based on cumulative DNA damage (the area under the TMZ-induced damage curve), and 3) the extent of variation of MGMT protein over the course of the day approximately agrees with the variation in the *Mgmt* gene (10-fold difference between maximum and minimum).

To handle the unknowns around the parameters reflecting the relative strengths of TMZ vs MGMT, we simulate a wide range of potential values. As our goal is to understand what dosing times are optimal given a fixed MGMT protein waveform (peaking at CT4) for parameters that best match the available data, the quality of the model fit to data from [14] is determined using the mean cell death induced by TMZ across all times as well as the range of variation (maximum - minimum cell death) between the simulation results and the data, but not the specific cell death values at a given *time* of day. Thus, we ignore all time information in our fitting process, and seek to determine if peak and trough times consistent with previously published data [14] can be recovered with our mathematical model.

## Results

### GBM has daily rhythms in MGMT protein expression and promoter methylation

**Fig. 1:**
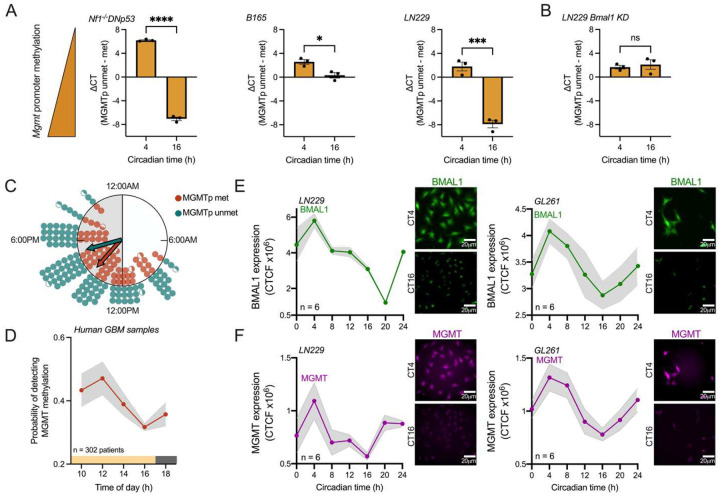
GBM exhibits daily rhythms in MGMT promoter methylation and protein abundance. A) Murine Nf1^−/−^ DNp53, primary B165, and human LN229 GBM cells show daily rhythms in *Mgmt* promoter methylation *in vitro*, with lower levels observed at Circadian time 4 (CT4), and higher methylation observed at CT16 (n=3 samples per time point, mean±SEM, Student’s t test, *p < 0.05, ***p < 0.001, ****p < 0.0001). B) Human LN229 cells lacking a functional circadian clock (LN229 *Bmal1* KD) show no daily rhythms in *Mgmt* promoter methylation *in vitro*, with both CT4 and CT16 time points showing low methylation levels (n=3 samples per time point, mean±SEM, Student’s t test, ns p > 0.05). C) Distribution of patients with GBM tumors scored as MGMT promoter methylated (MGMTp met, orange circles) or unmethylated (MGMTp unmet, blue circles) as a function of time-of-day of biopsy. In these Rayleigh plots, each filled circle equals data from two patients and half-circle from one patient, each arrow points to the mean time of day of the samples and the length of the arrow indicates the variation from the mean phase (0=random, 1=all patients methylated or unmethylated at the same time of day). D) The probability of a tumor biopsy being identified as MGMTp methylated is higher around noon, and reaches a minimum around 4:00pm (n=302 patients, annual mean±SEM over 5 years of tumor collection, average trace scored circadian by JTK cycle, p < 0.05). E-F) LN229 (left) and GL261 (right) cells show daily rhythms in BMAL1 (E, green) and MGMT (F, purple) protein expression, peaking at CT4 in both cell lines. Representative images of staining for BMAL1 and MGMT at CT4 (peak) and CT16 (trough) are shown (n=6 samples per time point, mean±SEM, scale bar = 20 μm, average trace scored circadian by JTK cycle, p < 0.05).

A recent study found that LN229 GBM cells have intrinsic circadian rhythms in sensitivity to TMZ and in *Mgmt* promoter methylation, which controls the expression of the DNA repair enzyme MGMT [14]. Yet it is unknown whether other GBM models also show daily rhythms in *Mgmt* promoter methylation. To test the hypothesis that MGMT promoter status varies with time of day in GBM cells of different backgrounds, we first assessed whether *Mgmt* promoter methylation varies with circadian time in murine (Nf1^−/−^DNp53), human (LN229), and primary (B165) GBM cells *in vitro*. Across all GBM cell lines, we found lower *Mgmt* promoter methylation when cells were collected in the subjective morning, at Circadian time 4 (CT4), and higher in the subjective evening, at CT16 (Figure 1A). CT was previously defined relative to expression of the clock gene *Per2*, where CT4 and CT16 corresponds to 4h and 16h after peak *Per2* expression, respectively [14, 17]. To test if daily rhythms in *Mgmt* promoter methylation depend on an intact circadian clock, we measured *Mgmt* promoter methylation in LN229 cells lacking the core clock gene *Bmal1* (LN229 *Bmal1* KD), which have been previously shown to have disrupted circadian rhythms [14, 17]. We found that LN229 *Bmal1* KD cells show no daily rhythms in *Mgmt* promoter methylation, with methylation levels being low at both circadian times (Figure 1B), suggesting that the circadian clock regulates daily methylation of the *Mgmt* promoter.

To determine if daily rhythms in *Mgmt* promoter methylation impact diagnosis across the heterogeneity of GBM tumors, we next tested whether detecting *Mgmt* promoter methylation changes with time of collection in patient biopsies. We evaluated *Mgmt* promoter status from patient biopsies collected at different times of day, from 10am to 6pm, binned every 2 hours. We analyzed *Mgmt* promoter status annually from a total of 302 GBM patients who underwent surgery between 2020–2024 (i.e., 5 patient cohorts separated by year of biopsy). We found the number of samples scored as *Mgmt*-methylated peaked in biopsies collected around midday (Figure 1C). We further found that the probability of detecting *Mgmt* promoter methylation among all patient GBM samples showed a daily rhythm that peaked around noon (Figure 1D, average trace scored circadian by JTK cycle, p < 0.05), varying by at least 20% over the course of the day.

Finally, we evaluated whether GBM cells have daily rhythms in MGMT protein abundance. We used the well-characterized human LN229 and murine GL261 GBM cells, which have been previously found to have reliable circadian rhythms in clock gene expression and response to TMZ chemotherapy [14, 17]. We fixed GBM cells every 4 hours over 24 hours and performed immunostaining for MGMT and BMAL1 proteins (Figures S1 and S2). In both human and murine GBM cell lines, we found daily rhythms in expression of the clock protein BMAL1 (Figure 1E) and MGMT (Figure 1F) peaking during the subjective morning, at CT4 (average traces scored circadian by JTK cycle, p < 0.05). The amplitude and phase of these protein rhythms are consistent with a previous report of *Mgmt* and *Bmal1* mRNA and promoter methylation rhythms in GBM cells *in vitro* [14]. We conclude that *Mgmt* methylation status and MGMT abundance in GBM varies with time of day of sample collection.

### Mathematical modeling predicts maximal TMZ efficacy in the early subjective morning

As CT4, the time of peak MGMT protein abundance, was the time corresponding to maximal TMZ sensitivity in previous work [14], we sought understand these counterintuitive results through a mathematical model. We incorporated the daily rhythms in *Mgmt* promoter methylation and protein abundance into a system of differential equations capturing *in vitro* cell dynamics to predict the time of day when TMZ efficacy would be maximized given rhythmic MGMT activity in GBM. The conversion of TMZ to its toxic form, MTIC, in the nucleus was achieved using a previously published *in vitro* model [19]. We adapted this established *in vitro* model to incorporate daily rhythms in MGMT, where dynamics are driven by *k*_1_, which represents the strength of TMZ (i.e., how effectively it damages DNA), and *k*_2_, which represents the strength of MGMT (i.e., how effectively it repairs DNA).

**Fig. 2:**
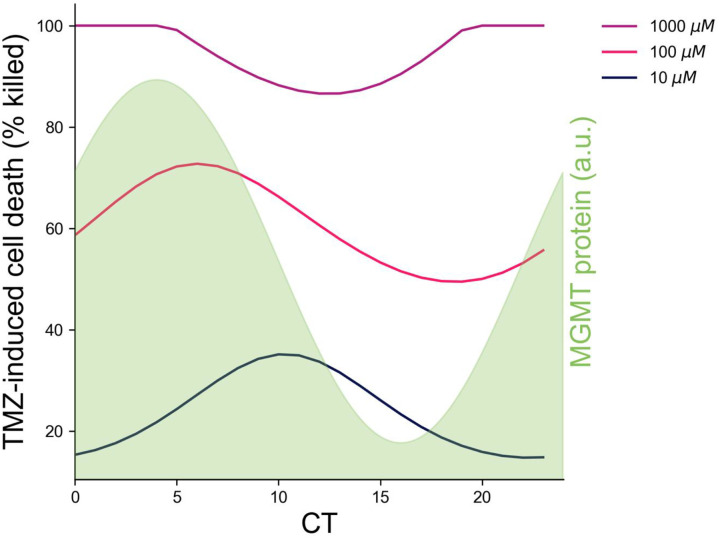
Efficacy of TMZ-induced cell death depends on dose and time after the daily peak of the MGMT protein. A mathematical model predicted the percent of GBM cell death *in vitro* varies following TMZ delivery at different circadian times (CT). Efficacy of 100 μM TMZ (pink line) showed a higher amplitude daily rhythm compared to 10 μM (black line) and 1000 μM (purple line) TMZ, with a peak following the maximum in MGMT protein abundance (green shaded background). TMZ induced around 73% cell death when delivered shortly following maximum MGMT abundance (CT6), while delivering when MGMT levels began to rise (CT16) induced around 49% cell death.

Using values for *k*_1_, and *k*_2_ which best fit the mean and range of variation of the data in [14] (*k*_1_ = 12.54, *k*_2_ = 0.818, *and d D*_*T*_ = 344), the model predicted that 73% cell death would occur with TMZ treatment around CT4–6 (i.e., subjective early morning), when MGMT abundance begins to decline, while 49% cell death would occur when treated at CT16–20 (i.e., subjective evening), as MGMT abundance begins to rise (Figure 2), in a dose-dependent manner. Nearly identical results were seen for a simpler model (Fig. S4), while the same best time with even greater time-of-day variation was observed when a Michaelis-Menten term was added to the *in vitro* cell model (Figure S7). In all incarnations, the models predicted that 10 μM and 1000 μM TMZ would both have smaller time-of-day variations in efficacy than 100 μM, with 10 μM TMZ having reduced time-of-day variation due to the fact that it causes less DNA damage than 100 μM at all times, while 1000 μM TMZ reaches a threshold where *all* cells are killed, regardless of time of day (Figures 2, S4, S7).

**Fig. 3:**
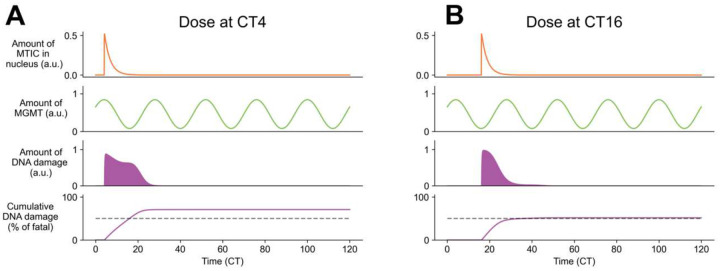
TMZ induces DNA damage and cell death as a function of the daily rhythm in MGMT protein abundance. A) *In vitro* mathematical modeling that incorporates daily rhythms in TMZ delivery (first row) and MGMT protein abundance (second row) predicted higher DNA damage (third row) and cumulative cell death (fourth row) when TMZ was delivered at CT4. The model predicted that TMZ at CT4, but not CT16, induced cumulative DNA damage that exceeded a 50% threshold (dashed line), leading to maximum TMZ-induced GBM cell death. B) Dosing at CT16, which corresponds to lowest MGMT protein abundance, yielded less DNA damage and cell death. The amount of predicted DNA damage lasted for a shorter time and barely crossed a 50% threshold, leading to reduced TMZ-induced GBM cell death. The top row in each plot shows the time profile of the toxic form of TMZ (MTIC) in the nucleus, the second row shows daily MGMT protein, the third shows the damage to the DNA, and the fourth shows cumulative damage to the DNA, scaled by the fatal threshold *D*_*T*_. Parameters were *k*_1_ = 12.54, *k*_2_ = 0.818, and *D*_*T*_ = 344.

Using the same parameters that best fit the data in [14], we found that the time of TMZ arrival at the nucleus influenced the time course of DNA damage, with more cumulative DNA damage occurring when TMZ was delivered near or shortly after the daily maximum of MGMT and BMAL1 protein expression (CT4, Figure 3A), compared to when MGMT was at its daily minimum (CT16, Figure 3B). Together, these results suggest that an intermediate dose of TMZ timed according to daily rhythms in MGMT abundance in GBM results in greater cell death and maximum chemotherapy efficacy *in vitro*.

**Fig. 4:**
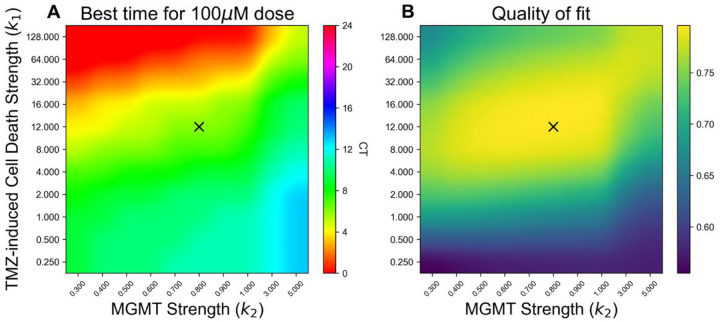
Early morning treatment with TMZ maximized efficacy and cell death, regardless of TMZ and MGMT strength. A) Modeling the *in vitro* strength of TMZ-induced DNA damage (y-axis) relative to the daily ability of MGMT to repair DNA (x-axis) showed that, across all the parameter sets tested that corresponded to high quality fits (yellow regions in B), the dose time that maximized cell damage was near the peak of MGMT (CT4) or on its descent, corresponding to subjective morning dosing. No optimal TMZ dosing times were found near the lowest levels of MGMT expression (dark blue, purple in color bar). B) Heatmap showing which parameter choices best matched the average DNA damage for different doses and the extent of dosing time variation in previously reported data [14]. Yellow indicates a closer fit to the data, while blue indicates a poor fit. Across all the parameter sets tested, optimal TMZ dosing was found with a subjective morning treatment. The X marks in A and B correspond to the parameter set that best matches the mean cell death and range of cell death observed in experimental data following 100 μM TMZ treatment [14], with best fit at CT6.

When we further tested the robustness of the optimal time for TMZ treatment with different choices of TMZ and MGMT rate constants, *k*_1_ and *k*_2_, (solving for *D*_*T*_ to match previously observed data from [14] for each *k*_1_, *k*_2_ pair), we found that the best time to dose with TMZ ranged from CT0 to CT12, with no optimal times occurring near MGMT protein’s lowest expression at CT16 (Figure 4A), despite the fact that dosing near MGMT protein’s minimum seems intuitively like an ideal time to dose. The parameters which best fit the data (Figure 4B, higher values in yellow correspond to better fit), corresponded to maximum effect from TMZ dosing between CT2 and CT8, agreeing with prior findings that maximum TMZ-induced tumor cell death occurs at CT4, around the time when daily BMAL1 and MGMT protein levels begin to decrease, but not when they reach their lowest abundance. Results from a second model with fewer assumptions yielded similar results (Figures S3-S6), as did a version of the model with a Michaelis-Menten term added (Figures S7-S9), indicating that 100 μM TMZ dosing as daily MGMT protein abundance begins to decrease provides maximal efficacy against GBM growth.

In summary, our model predicted that TMZ efficacy on GBM cells *in vitro* peaks as MGMT protein decreases. By extrapolation, based on the time of peak BMAL1 expression in mouse xenograft models of GBM [14, 17], we predict TMZ should be given around CT4, corresponding to treatment in the subjective early morning. When delivered in the subjective morning as MGMT abundance declined, a 100 μM dose of TMZ induced an additional 25–35% GBM cell death compared to delivering in the subjective afternoon/evening when MGMT abundance peaked (Figures 2, S4, S7).

### GBM cells are more sensitive to TMZ when delivered at earlier circadian times

**Fig. 5:**
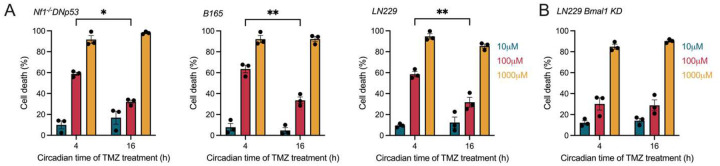
GBM TMZ sensitivity depends on circadian time of treatment *in vitro*. A) Murine Nf1^−/−^ DNp53, primary B165, and human LN229 GBM showed higher cell death when treated with 100 μM TMZ (red bars) at CT4 compared to CT16. No daily rhythm was observed when treating cells with 10 (blue bars) or 1000 (yellow bars) μM TMZ (n = 3 per group, mean±SEM, two-way ANOVA with Šídák’s multiple comparisons test, *p < 0.05, **p < 0.01). B) LN229 cells lacking a functional circadian clock (LN229 *Bmal1* KD) had dose-, but not time-of-day, dependent responses to TMZ treatment (n = 3 per group, mean±SEM, two-way ANOVA with Šídák’s multiple comparisons test, ns p > 0.05).

To validate the predictions of our *in vitro* mathematical models, we treated murine (Nf1^−/−^DNp53), human (LN229), and primary (B165) GBM cell lines with three different doses of TMZ at either the predicted optimal (CT4) or suboptimal (CT16) circadian times. We found that TMZ dose-dependently induced cell death, and dependent on circadian time, consistent with the mathematical models. Specifically, we found that 100 μM TMZ induced an average of 60% cell death when delivered at CT4, compared to 30% at CT16, in all GBM cells *in vitro* (Figure 5A). The daily rhythm in TMZ sensitivity was not detected at 10 or 1000 μM TMZ, with 10 μM inducing less than 10% and 1000 μM inducing 90% cell death. We next assessed if daily rhythms in sensitivity to TMZ depend on an intact circadian clock by treating LN229 *Bmal1* KD cells with TMZ at either CT4 or CT16. We found that knocking down *Bmal1* abolished the daily rhythm in TMZ sensitivity, with all doses yielding similar percentages of cell death regardless of time of day of treatment (Figure 5B). We conclude that GBM sensitivity to TMZ *in vitro* increases around CT4, corresponding to when daily MGMT and BMAL1 protein abundance begins to decline.

## Discussion

Our results suggest that, across multiple GBM cell lines, *Mgmt* promoter methylation levels change with time of day of collection, with lower methylation levels observed in the subjective afternoon *in vitro*. Consistent with this, we find that the probability of detecting *Mgmt* promoter methylation significantly decreases from around 50% to 30% when patients were biopsied later in the day compared to in the morning. This timing and amplitude aligns with the reported daily oscillations in *Mgmt* mRNA and promoter methylation in human GBM cells *in vitro* [14]. Taking into consideration the limitations of this retrospective analysis, future clinical trials should be designed to better control for variables like the time of sample fixation, as well as other comorbidities like patient age and sex. Further, to account for interpersonal differences in circadian rhythms, it will be important to measure how *Mgmt* promoter methylation and expression varies with time of day in individual GBM patients.

Daily rhythms in MGMT protein expression are likely driven by the circadian clock transcription factor, BMAL1, across the variety of GBM cells. We previously reported that *Mgmt* mRNA levels peak around 12 hours after peak *Bmal1* mRNA [14]. We now find that maximal BMAL1 protein aligns with the time of maximal transcription and translation of MGMT. We posit that BMAL1 promotes *Mgmt* expression, which is rapidly translated into MGMT protein. Altogether, these results suggest that cell-intrinsic circadian rhythms regulate MGMT abundance and promoter methylation so that scoring MGMT status on GBM biopsies varies with time of sample collection. Considering the clinical challenges posed by repeating biopsies at multiple times of day, we propose that future studies could employ approaches like focused ultrasound-enhanced liquid biopsies or *ex vivo* organotypic patient-derived slice cultures that can accurately and less-invasively measure daily MGMT expression and promoter methylation in individual patient samples [20, 21].

Our experimental data suggests, surprisingly, that the best time to dose with TMZ aligns with the peak of MGMT protein abundance, despite the fact that MGMT actively works against the effects of TMZ. To understand this counterintuitive result, we built a mathematical model of TMZ dynamics by extending a previously published *in vitro* model. Our model found that, across all parameters tested, it was never optimal to dose at the minimum of MGMT abundance, and that the parameter values which best captured the average cell death and range of cell deaths observed in [14] yielded CT2 - CT8 as the most effective times to dose with 100 μM TMZ. This result can be understood in the following way: TMZ does not act instantaneously but rather is cleared from the body in the hours after dosing. When the dose occurs at the minimum of MGMT, the exposure period overlaps with rising MGMT, and the drug-induced DNA damage is immediately undone by higher and higher MGMT levels. “Dosing on the way down” (i.e., when daily MGMT levels begin to decline) means that the exposure period of the drug overlaps with a period during which MGMT is declining and at its lowest values. This means that DNA damage done by the drug is left unrepaired by MGMT for a longer period of time, which acts to increase the area under the DNA damage curve and contribute to greater likelihood of cell death.

Consistent with previous studies that find robust daily rhythms in TMZ efficacy when delivered at 100 μM *in vitro* [14], we find that 100 μM TMZ has the highest amplitude daily rhythms, being most effective as MGMT levels fall from their daily peak. We find that this result holds true for all tested values, which include TMZ strength (*k*_1_), MGMT strength (*k*_2_), and threshold of DNA damage at which cell death occurs (*D*_*T*_). Lastly, we find that best time to dose depends on the amount of the drug being delivered, and that “windows of circadian effect”—where low doses show minimal circadian variation because of the noise floor while high doses show minimal circadian variation because of ceiling effects—can be explained with a theoretical model. Because our mathematical models include parameters that can be best simulated *in vitro*, future studies should assess which are the best times of day for TMZ delivery *in vivo* in different GBM xenografts and in human patients. Future studies should consider interactions between TMZ timing, survival, and side effects. Further, because the major effects of TMZ are observed when delivered concurrently with radiation, future studies should also assess whether daily sensitivity to TMZ is changed under chemoradiation paradigms to better model the standard of care and potential translation of these findings into the clinic.

Previous studies find that maximum *Mgmt* methylation occurs at CT16 in GBM cells [14]. We now find that *Mgmt* promoter methylation peaks 12 hours after peak MGMT protein. Given that *Mgmt* promoter methylation appears to peak around noon in human biopsies, we estimate that MGMT protein will peak in the middle of the night. Our modeling results suggest that dosing near the peak of MGMT and as it begins its decrease is a better strategy, compared to dosing near its trough. This suggests that the benefit to morning dosing seen in retrospective human data may have been driven primarily by patients who dosed in the early morning.

One limitation of this study is that we do not know the intratumoral concentration of TMZ when delivered at different circadian times. Previous studies have modeled TMZ pharmacokinetics and found that peak concentrations ranged from 14 to 35 μM to in human GBM, which best match clinically relevant doses (i.e., 75–200 mg/m^2^/day) [22]. While we find higher amplitude daily rhythms in sensitivity to TMZ with a 100 μM dose, one important finding is that lower doses like 10 μM also show a daily rhythm with peak sensitivity later in the early morning, closer to typical waking time. This suggests that clinically relevant doses can also be timed to the morning to obtain maximum chemotherapy efficacy. Future studies should measure the intratumoral concentration of TMZ when delivered at different circadian times and in combination radiation to further determine the optimal times to dose in the clinic. Altogether, our results support a model whereby dosing near the minimum of MGMT protein abundance means dosing right when repair mechanisms are gearing up again, blunting the effect of TMZ, while dosing near the maximum of MGMT or on the way down is equivalent to dosing at the start of a window of vulnerability when MGMT is lowered for a prolonged period and repair mechanisms are more quiescent.

Our findings have significant implications for both chronodiagnosis and chronomodulation of therapies for GBM. While *Mgmt* methylation may be thought of as a binary measurement, where methylation leads to gene silencing and unmethylation leads to increased MGMT expression, our results suggest that this process may be more dynamic and, in part, regulated by the circadian clock. If there exists, for instance, a daily *Mgmt* methylation rhythm across individuals, with some having low or high methylation levels, it may be that the only individuals being diagnosed as *Mgmt* methylated are those with sufficiently high amplitude expression levels and who are biopsied near the *Mgmt* methylation peak. Clinically, this may suggest that many individuals may be mischaracterized as having an unmethylated or methylated tumor because of the time of day when samples were collected, which could in turn affect prognosis and optimal treatment strategies to deliver chemotherapy with TMZ.

Recent studies argue that patients with MGMT-unmethylated GBM tumors show limited benefit of TMZ but have not considered circadian time as an important variable for both measuring *Mgmt* methylation and assessing TMZ efficacy [11]. A previous study found that delivering TMZ in the morning yields a 6-month increase in overall survival, specifically for patients diagnosed with MGMT methylated GBM tumors [13]. However, whether these patients exhibited a daily rhythm in MGMT methylation that underlied the increased response to TMZ in the morning remains unknown. We now find that MGMT methylation diagnoses in humans vary by at least 20% (i.e., 50% when biopsied early in the day, and 30% when collected later in the day). This has both significant clinical and therapeutic implications. First, patients biopsied when the rhythm in *Mgmt* methylation is low (during the late afternoon or night) may be getting a diagnosis of MGMT-unmethylated glioma even though they would have been diagnosed as an MGMT-methylated glioma with another biopsy time. This diagnosis is essential to determine the prognosis and therapeutic approach a patient will receive, and thus can be a source of undue stress for patients and their families when weighing potential treatments. Second, treating MGMT unmethylated tumors has remained an elusive goal. Our findings suggest that if *Mgmt* methylation status is more of a dynamic process, regulated by the circadian clock, than a fixed and unchanging characteristic of the tumor, then therapeutically targeting the circadian clock may be an effective approach to boost daily rhythms in *Mgmt* methylation and increase its silencing at key times of the day. Future work should test if daily rhythms in *Mgmt* methylation vary within individuals and whether the daily amount of *Mgmt* methylation underlies TMZ efficacy in GBM patients.

Chronotherapy, also known as circadian medicine or chronomedicine, has proven effective in treating acute lymphoblastic leukemia, colorectal, ovarian, and other gynecological cancers, and more recently GBM [13, 23–27]. However, it is not yet the standard of care nor has it been thoroughly evaluated in many cancers. The potential to enhance treatment outcomes for approved drugs—supported by evidence demonstrating improved survival rates and reduced side effects across various cancer types—highlights the importance of further exploring personalized chronotherapy as a therapeutic strategy for cancer treatment. Additionally, discovering new cancer treatment drugs remains crucial, and future research will benefit from tracking the time of day of treatment and the chronotype of individual patients. Our findings support that *in vitro* mathematical modeling that incorporates daily fluctuations in gene and protein expression can predict the optimal times to deliver anticancer drugs to obtain maximum efficacy. These findings may be generalizable to other systems of interest—in particular, in situations where the drug has a relatively short half-life, like TMZ, and the goal is to maximize the integral of some interaction between the drug and its target. Future work could target personalizing TMZ timing to each individual, using real-world circadian tracking interventions that incorporate modeling of daily rhythms in GBM tumors, in order to maximize TMZ efficacy and ultimately improve patient outcomes.

## Supplementary Files

This is a list of supplementary files associated with this preprint. Click to download.

- SupplementaryMaterials.docx

## Data Availability

All data and code reported in this paper will be shared upon request by the corresponding authors.
